# Rotations with Indian Mustard and Wild Rocket Suppressed Cucumber Fusarium Wilt Disease and Changed Rhizosphere Bacterial Communities

**DOI:** 10.3390/microorganisms7020057

**Published:** 2019-02-21

**Authors:** Xue Jin, Jian Wang, Dalong Li, Fengzhi Wu, Xingang Zhou

**Affiliations:** 1 Key Laboratory of Biology and Genetic Improvement of Horticultural Crops (Northeast Region), Ministry of Agriculture, Northeast Agricultural University, Harbin 150030, China; 497791690@163.com (X.J.); lilong00000@163.com (D.L.); wufz@neau.edu.cn (F.W.); 2 Department of Horticulture, Northeast Agricultural University, Harbin 150030, China; z220wangjian@163.com

**Keywords:** bacterial community composition, crop rotation, *Cucumis sativus* L., fusarium wilt, *Pseudomonas* spp

## Abstract

Crop monocropping usually results in an enrichment of soil-borne pathogens in soil. Crop rotation is an environmentally friendly method for controlling soil-borne diseases. Plant rhizosphere microorganisms, especially plant-beneficial microorganisms, play a major role in protecting plants from pathogens, but responses of these microorganisms to crop rotation remain unclear. Here, we evaluated the effects of rotations with Indian mustard (*Brassica juncea*) and wild rocket (*Diplotaxis tenuifolia* (L.) DC.) on cucumber Fusarium wilt disease caused by *Fusarium oxysporum* f.sp. *cucumerinum* (FOC). Cucumber rhizosphere bacterial community composition was analyzed by high-throughput amplicon sequencing. Bacteria, *Pseudomonas* spp., 2,4-diacetylphloroglucinol (an antifungal secondary metabolite) producer and FOC abundances were estimated by real-time PCR. Rotations with Indian mustard and wild rocket suppressed cucumber Fusarium wilt disease and cucumber rhizosphere FOC abundance. Crop rotations increased cucumber rhizosphere bacteria, *Pseudomonas* spp. and 2,4-diacetylphloroglucinol producer abundances. Moreover, crop rotations changed cucumber rhizosphere bacterial community composition and increased bacterial community diversity. However, crop rotations decreased soil inorganic nitrogen content and inhibited cucumber seedling growth. Overall, rotations with Indian mustard and wild rocket suppressed cucumber Fusarium wilt disease, which might be linked to the increased rhizosphere bacterial diversity and abundances of potential plant-beneficial microorganisms (such as *Pseudomonas* spp. and 2,4-diacetylphloroglucinol producer).

## 1. Introduction

Plant soil-borne diseases are a major cause of crop losses and are difficult to control with conventional strategies, such as the use of resistant host cultivars and synthetic fumigants [1]. Intensive agricultural systems based on crops grown in monoculture or short rotation are usually not sustainable in the long-term since these systems are vulnerable to plant diseases, which seriously threaten global food security [2,3]. It is generally known that agricultural systems that increase the spatial and temporal plant diversity in the field (*e.g*., intercropping, crop rotation and use of cover crop or green manure) can help to manage soil fertility, maintain physical soil properties, and prevent the build-up of soil-borne pathogens [1,3,4,5,6,7]. Crop rotation can suppress plant soil-borne diseases through several mechanisms such as interrupting the cycling of pathogens, releasing antifungal chemicals and changing soil microbial communities [3,5,7].

Plant rhizosphere microorganisms are key determinants of plant health and productivity, and are considered to be a major driver of plant defense to belowground pathogens [8]. In particular, plant-beneficial microorganisms (such as some species in *Pseudomonas* spp.) can protect plants directly by inhibiting plant pathogens and indirectly by inducing systemic resistance in plants through their secondary metabolites (such as 2,4-diacetylphloroglucinol, DAPG) [9,10]. In crop rotation systems, the proceeding crop can change soil chemical properties (such as soil nutrient contents) and therefore affect the physiological status (such as root exudation) of the succession crop [4,11]. Soil microorganisms rely on carbon and nutrient resources from plant rhizodepositions and litters [12,13]. The chemistry compositions of rhizodepositions and litters differ among plant species, and, thus, plants can exert species-specific effects on soil microbial communities [12,13,14]. Numerous studies have observed that crop rotation can increase soil microbial diversity and activity, and change soil microbial community composition [4,5,7,15,16]. However, the responses of specific potential plant-beneficial microorganisms and their disease suppressive functional genes to crop rotation remains unclear.

It has been shown that green manuring, the practice of incorporating actively growing plant materials into soil, can suppress soil-borne pathogens and diseases of several crops [1]. For example, green manures of Brassicaceae crops, such as Indian mustard (*Brassica juncea*) and wild rocket (*Diplotaxis tenuifolia* (L.) DC.), could inhibit pathogenic *Fusarium* spp. and control Fusarium wilt of cucumber (*Cucumis sativus* L.), spinach (*Spinacia oleracea* L.) and tomato (*Solanum lycopersicum* L.) [17,18,19]. Green manures of Brassicaceae crops can inhibit plant soil-borne pathogens directly by releasing antifungal compounds (e.g., isothiocyanates) and indirectly by changing soil microbial communities [1,20]. However, no profitable aboveground material was harvested from these green manure crops, which may discourage farmers to use this practice. There is also evidence showing that rotations with Brassicaceae crops, in which aboveground materials of Brassicaceae crops are harvested and not incorporated into soil, can suppress plant soil-borne diseases [21,22,23].

Fusarium wilt is a vascular soil-borne disease worldwide and can cause severe damage to many economically important crops [24]. Fusarium wilt of cucumber (*Cucumis sativus* L.), caused by *Fusarium oxysporum* f.sp. *cucumerinum* (FOC), is one of the major factors limiting cucumber production [6]. Previously, we observed that green manures of Indian mustard and wild rocket suppressed cucumber Fusarium wilt disease. In this study, we further assessed the effects of rotations with Indian mustard and wild rocket, in which aboveground biomasses were removed and not incorporated into soil, on cucumber Fusarium wilt disease and rhizosphere bacterial communities. It has been observed that increasing plant diversity in the field can stimulate plant-beneficial microorganisms in soil, such as *Pseudomonas* spp., and thus inhibit soil pathogens [5,20,25]. We hypothesized that rotations with Indian mustard and wild rocket could decrease Fusarium wilt disease, change microbial community composition and enhance plant-beneficial microorganisms in cucumber rhizosphere. In this study, cucumber rhizosphere microbial communities were analyzed by real-time PCR and high-throughput amplicon sequencing.

## 2. Materials and Methods

### 2.1. Soil Preparation

Soil used in this study was collected from the upper soil layer (0–15 cm) of a greenhouse in the experimental station of Northeast Agricultural University, Harbin, China (45°41′N, 126°37′E). The greenhouse has been cultivated continuously with cucumber since 2006. Two croppings of cucumber (cv. Jinyan 4, Institute of Vegetable Crops, Tianjin, China) were cultivated in each year, one in spring (April to July) and the other in autumn (July to October). Soil sampling was performed in July 2016 after the harvest of the first cropping of cucumber. Soils were sieved (2 mm), and large stones and plant derbies were removed. The soil was sandy loam, contained organic matter, 3.51%; inorganic N (NH_4_^+^-N and NO_3_^−^-N), 146.60 mg/kg; Olsen P, 284.20 mg/kg; available K, 341.80 mg/kg; EC (1:2.5, *w*/*v*), 0.43 mS/cm; and pH (1:2.5, *w*/*v*), 7.64.

### 2.2. Pot Experiment

A pot experiment was conducted from July to September 2016. Indian mustard (cv. Xuelihong) and wild rocket (cv. Shuangji) were directly seeded into plastic pots (20 cm in diameter, 17 cm in height) containing 2.5 kg fresh soils prepared as above. There were 30 seeds per pot. After emergence, Indian mustard and wild rocket seedlings were thinned to ten plants per pot. A fallow treatment, pots with no Indian mustard or wild rocket cultivated, was served as control. Therefore, there were three treatments in total, pots cultivated with Indian mustard and wild rocket, and a fallow treatment. No fertilizer was added during the experiment. All pots were maintained in a greenhouse (32 °C day/22 °C night, relative humidity of 60–80%, 16 h light/8 h dark). Each treatment included three replicates (i.e., three blocks) with 20 pots per replicate (60 pots per treatment in total). The position of these pots in each block was randomly changed every three days. Soil water content was adjusted every two days with distilled water to maintain the soil moisture at about 65% of its water holding capacity.

Forty days after seeding, the above ground materials of Indian mustard and wild rocket were harvested by cutting at the soil line with a scissor. All aboveground material was removed from the pots and not incorporated into soil. Roots of Indian mustard and wild rocket were left in the soil. Then, all pots were covered with black polyethylene films and incubated for 30 days with soil water content maintained at about 65% of its water holding capacity.

After incubation, cucumber seedlings were planted in the pots with rotation crops and the fallow treatment. Cucumber seeds (cv. Jinyan 4, susceptible to FOC) were soaked in water at 55 °C for 30 min and then germinated in sand in a growth chamber at 25 °C. After emergence, cucumber seedlings were transplanted into the above pots. Each pot contained one seedling. All pots were maintained in a greenhouse (32 °C day/22 °C night, relative humidity of 60–80%, 16 h light/8 h dark). Soil water content was adjusted every two days with distilled water to maintain the soil moisture at about 65% of its water holding capacity. For these cucumber seedlings, 15 plants in each treatment per replicate were inoculated FOC to monitor Fusarium wilt disease severity. Other non-inoculated seedlings (five plants) were used to measure plant dry biomass and collect rhizosphere soils.

### 2.3. Inoculation of FOC and Fusarium Wilt Disease Severity Measurement

The FOC strain (race 4) used was isolated and identified from a Fusarium-wilted cucumber plant grown in a greenhouse. FOC was grown on potato-dextrose-agar (PDA) medium and conidia were obtained as previously described [26]. Fifteen days after transplanting, cucumber seedlings at the two-leaf stage were inoculated with FOC with a root-dipping method as previously described [27]. Briefly, cucumber seedlings were removed from soil and washed with sterile water. Then, root tips were cut off with a sterilized scissor and dipped in a FOC conidial suspension (2 × 10^8^ conidia mL^−1^) for 10 min. Afterwards, these inoculated cucumber seedlings were transferred back to their original pots. Fifteen days after the inoculation of FOC, the Fusarium disease severity of cucumber seedlings expressed as Fusarium wilt disease index was recorded and calculated using a scale containing six grades as suggested by Liu et al. [26].

### 2.4. Plant Dry Biomass Measurement and Soil Sampling

Thirty days after transplanting, noninoculated cucumber seedlings were harvested and the plant dry biomass was measured after oven drying at 70 °C to constant weight. Meanwhile, bulk soil and cucumber rhizosphere soil samples were collected as previously described [28]. Samples from ten plants (for rhizosphere soils) or pots (for bulk soils) in each replicate of the individual treatment were combined to make a composite sample. After sieving (2 mm mesh), these fresh bulk soils were used for soil chemical analysis while rhizosphere soils were stored at −80 °C for DNA extraction.

### 2.5. Soil Chemical Analysis

Soil pH was determined with 10 g soil in water suspensions at a soil/water ratio of 1:2.5 with a glass electrode. For soil inorganic N and Olsen P, soils (10 g) were extracted with 0.5 M sodium bicarbonate and 2 M potassium chloride, respectively, and were determined with a continuous flow analyzer (San^++^, SKALAR, Netherlands). Soil phenolic compounds were extracted from 15 g soil with 2 M NaOH and measured by the Folin-Ciocalteau method expressed as μg of ferulic acid equivalents per gram of soil dry-weight [29].

### 2.6. Soil DNA Extraction

Total soil DNA was extracted from 0.25 g of each individual rhizosphere soil sample with the PowerSoil DNA Isolation Kit (QIAGEN, Venlo, the Netherlands) as per the manufacturer’s instructions. Electrophoresis in a 1.2% (*w*/*v*) agarose gel stained with ethidium bromide was performed in order to check the yield and quality of the extractions. Each composite soil sample was extracted in triplicate and the extracted DNA solutions were pooled. There were three composite DNA solution samples for each treatment.

### 2.7. Quantitative PCR Analysis

Cucumber rhizosphere total bacteria, *Pseudomonas* spp., DAPG producer and FOC abundances were estimate by SYBR Green quantitative PCR assays conducted with an IQ5 real-time PCR system (Bio-Rad Lab, Hercules, CA, USA). For total bacteria and *Pseudomonas* spp., primer sets of 338F/518R [30] and PsF/PsR [31] were used to amplify the partial bacterial 16S rRNA genes. For the DAPG producer, the primer set of B2BF/B2BR3 [32] was used to amplify the gene *phlD* that was responsible for DAPG production. For FOC, a FOC-specific SCAR primer set FocF8/FocR2 [33] was used. The PCR protocols were: 95 °C for 5 min; followed by 35 cycles of 95 °C for 30 s, 56 °C for 45 s for 338F/518R (65 °C for 30 s for PsF/PsR; 67 °C for 30 s for B2BF/B2BR3; 65 °C for 30 s for FocF8/FocR2), 72 °C for 45 s; and a final extension at 72 °C for 5 min. The 20 μL PCR mixture contained 10 μL of 2 × TransStart SYBR Green qPCR SuperMix (Transgen Biotech, Beijing, China), 0.2 mM of each primer, 2.5 ng of soil DNA. Standard curves were created with 10-fold dilution series of plasmids containing the target genes. The threshold cycle (*C*t) values obtained for each sample were compared with the standard curve to determine the initial copy number of the target gene. Sterile water was used as a negative control to replace the template. All amplifications were performed in triplicate. The specificity of the products was confirmed by melting curve analysis and agarose gel electrophoresis.

### 2.8. High-Throughput Amplicon Sequencing and Data Processing

Cucumber rhizosphere bacterial community composition was analyzed with high-throughput amplicon sequencing. The V4–V5 regions of the bacterial 16S rRNA gene were amplified with primer set of F515/R907 on an ABI GeneAmp^®^ 9700 PCR System (ABI, Waltham, MA, USA) as previously described [27,34]. To distinguish each sample, both the forward and reverse primers had a six-bp unique barcode. Three technically replicated PCR reactions were performed for each composite soil DNA as suggested before [35], and PCR products from each composite soil DNA were pooled. Then, these pooled PCR products were purified with an Agarose Gel DNA purification kit (TaKaRa, Dalian, China) and quantified with a TBS-380 micro fluorometer (Invitrogen, Waltham, MA, USA). Equimolar amounts of these purified PCR products were pooled together and sequenced on an Illumina Miseq platform (2 × 300) at Majorbio Bio-Pharm Company (Shanghai, China).

The raw sequence data were de-multiplexed, quality-filtered, and processed using FLASH [36] as previously described [4]. The high-quality sequences were used to generate operational taxonomic units (OTUs) at 97% sequence similarity with UPARSE [37]. Then, the taxonomic information of a representative sequence from each phylotype was determined with the Ribosomal Database Project database [38]. Chimeric sequences were identified and removed using USEARCH 6.1 in QIIME [39]. To correct the sampling effort, randomly subsampled 20,445 16S rRNA gene sequences per sample were used for subsequent community analysis. All sequences have been deposited in the NCBI-Sequence Read Archive (Accession Number SRP180383, accessed on 18 January 2019).

### 2.9. Statistical Analysis

Bacterial community alpha diversity indices, including Good’s coverage, ACE Chao, Shannon index and inverse Simpson index were generated using QIIME [39]. The defined OTUs were used to calculate taxon accumulation curves. For beta diversity, bacterial community composition was analyzed using principal coordinates analysis (PCoA) based on the Bray-Curtis distance dissimilarity. Three different complementary non-parametric multivariate statistical tests, including analysis of similarities (ANOSIM), non-parametric multivariate ANOVA (adonis), and multiple response permutation procedure (MRPP) analyses were used to test the differences in bacterial communities with the Bray-Curtis distance and 999 permutations. Taxon accumulation curves, PCoA, ANOSIM, adonis and MRPP analyses were performed using with the “vegan” package in “R” (Version 3.3.1, R Foundation for Statistical Computing, Vienna, Austria).

All data were checked for normality (Shapiro-Wilk’s test) and homogeneity of variances (Levene’s test). Data of microbial abundances from quantitative PCR analysis were logarithmically transformed. Means were compared between treatments by the Tukey’s honestly significant difference (HSD) test. Differences were considered statistically significant at *p <* 0.05. Spearman’s rank correlations were calculated between cucumber seedling growth, Fusarium wilt disease severity and main bacterial taxa (average relative abundances >0.50% in at least one treatment) with the “psych” package in “R” (Version 3.3.1).

## 3. Results

### 3.1. Cucumber Fusarium Wilt Disease Severity and Seedling Biomass

Rotations with Indian mustard and wild rocket (with no incorporation of aboveground biomass) significantly decreased cucumber Fusarium wilt disease severity and cucumber seedling dry weight (*p <* 0.05) (Table 1). However, no difference in cucumber Fusarium wilt disease severity and seedling dry weight was observed between treatments of Indian mustard and wild rocket.

### 3.2. Soil Chemical Properties and Cucumber Rhizosphere Microbial Abundances

Rotations with Indian mustard and wild rocket had no influence on soil pH and Olsen P content but significantly decreased soil inorganic N and phenolic compounds contents (*p <* 0.05) (Table 1). Moreover, treatments of Indian mustard and wild rocket had similar soil inorganic N content.

**Figure 1:**
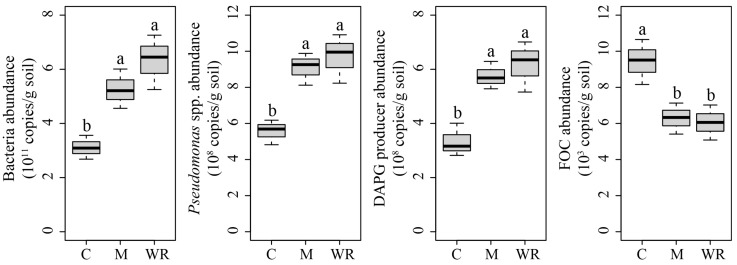
Cucumber rhizosphere bacteria, *Pseudomonas* spp. 2,4-diacetylphloroglucinol (DAPG) producer and *Fusarium oxysporum* f.sp. *cucumerinum* (FOC) abundances. C, M and WR represent treatments of cucumber monocropping, rotations with Indian mustard and wild rocket, respectively. Different letters indicate statistically significant differences among treatments (Tukey’s HSD, *p <* 0.05).

Rotations with Indian mustard and wild rocket significantly increased cucumber seedling rhizosphere total bacteria, *Pseudomonas* spp. and DAPG producer abundances but decreased FOC abundance (*p <* 0.05) (Figure 1).

### 3.3. Cucumber Rhizosphere Bacterial Community Alpha and Beta Diversities

**Figure 2:**
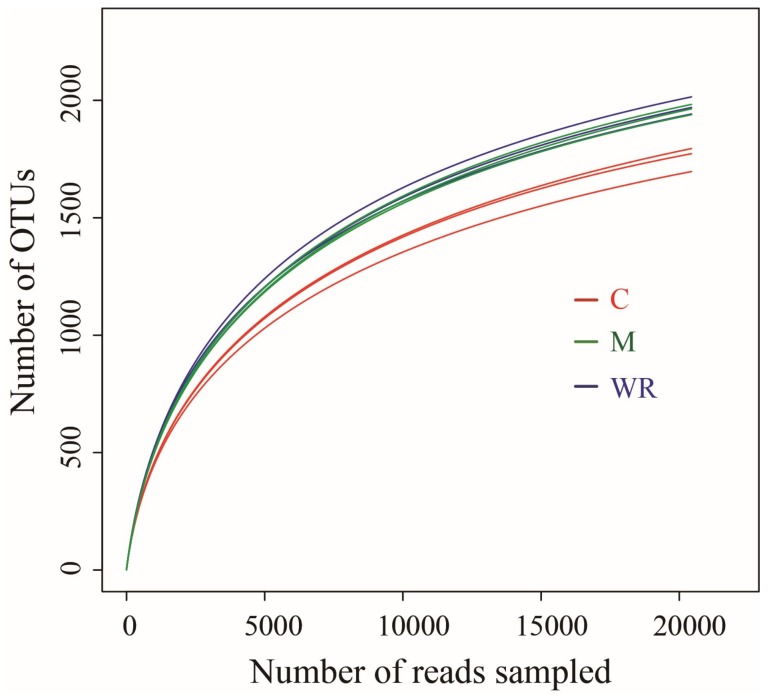
Rarefaction curves of the number of operational taxonomic units (OTUs) of cucumber rhizosphere bacterial communities. Random subsamples of 20,445 16S rRNA gene sequences per sample were used to generate the rarefaction curves. C, M and WR represent treatments of cucumber monocropping, rotations with Indian mustard and wild rocket, respectively.

In total, Illumina Miseq sequencing generated 235,875 quality bacterial 16S rRNA gene sequences with an average read length of 397 bp. The Good’s coverage of each sample, which reflects the captured diversity, was higher than 97.59% for all samples. Rarefaction curves of OTUs at 97% sequence similarity of all samples tended to approach the saturation plateau (Figure 2). Therefore, the sequencing depth was adequate for assessing the diversity of bacterial communities of our samples.

**Figure 3:**
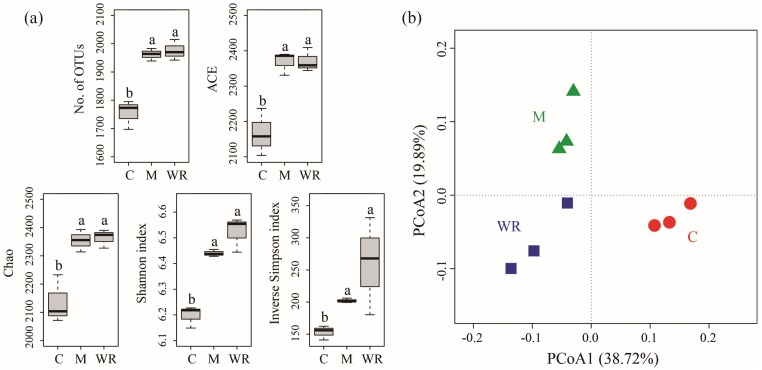
Cucumber rhizosphere bacterial community alpha (**a**) and beta diversities (**b**). C, M and WR represent treatments of cucumber monocropping, rotations with Indian mustard and wild rocket, respectively. Different letters indicate statistically significant differences among treatments (Tukey’s HSD, *p <* 0.05).

Rotations with Indian mustard and wild rocket significantly increased cucumber rhizosphere bacterial community alpha diversity indices (number of observed OTUs, ACE, Chao, Shannon and Inverse Simpson indices) (*p <* 0.05) (Figure 3a). Moreover, treatments of Indian mustard and wild rocket had similar cucumber rhizosphere bacterial community alpha diversity indices.

PCoA analysis revealed that samples from the same treatment grouped together, while different treatments were separated from each other (Figure 3b). Non-parametric multivariate statistical tests analyses demonstrated that cucumber rhizosphere bacterial community structure differed among treatments (ANOSIM, *R* = 0.909, *P* = 0.003; adonis, *R*^2^ = 0.545, *P* = 0.005; MRPP, Delta = 0.191, Effect size = 0.214, *P* = 0.004).

### 3.4. Cucumber Rhizosphere Bacterial Community Composition

**Figure 4:**
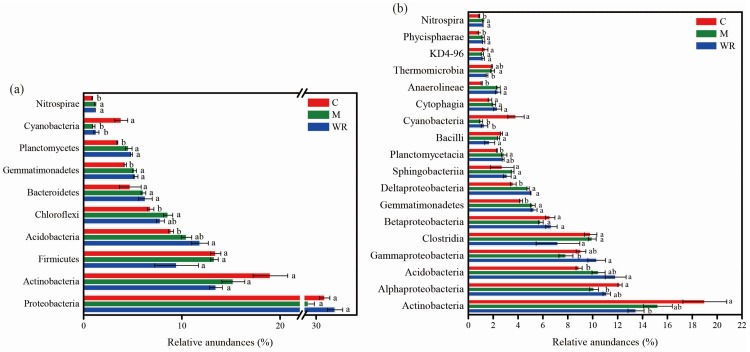
Relative abundances of main bacterial phyla (**a**) and classes (**b**) in cucumber rhizosphere. Bacterial phyla and classes with average relative abundances >1.0% in at least one treatment were shown. C, M and WR represent treatments of cucumber monocropping, rotations with Indian mustard and wild rocket, respectively. Different letters indicate statistically significant differences among treatments (Tukey’s HSD, *p <* 0.05).

In total, 35 bacterial phyla were detected, and 1.47% sequences were unclassified at the phylum level. Proteobacteria, Actinobacteria, Firmicutes and Acidobacteria were the dominant phyla (average relative abundances >10%) (Figure 4a). Rotations with Indian mustard and wild rocket significantly increased the relative abundances of Gemmatimonadetes, Planctomycetes and Nitrospirae, but decreased that of Cyanobacteria in cucumber rhizosphere (*p <* 0.05) (Figure 4a). Moreover, rotation with wild rocket increased the relative abundance of Acidobacteria, while rotation with Indian mustard increased the relative abundance of Chloroflexi in cucumber rhizosphere (*p <* 0.05).

At the class level, 85 bacterial classes were detected. Actinobacteria, Alphaproteobacteria, Acidobacteria, Gammaproteobacteria, Clostridia and Betaproteobacteria were the dominant classes (relative abundance >5%) (Figure 4b). Rotations with Indian mustard and wild rocket increased the relative abundances of Gemmatimonadetes, Deltaproteobacteria, Anaerolineae, Phycisphaerae and Nitrospira, but decreased that of Cyanobacteria in cucumber rhizosphere (*p <* 0.05) (Figure 4b). Moreover, rotation with Indian mustard decreased the relative abundance of Alphaproteobacteria and increased that of Planctomycetacia in cucumber rhizosphere (*p <* 0.05). Rotation with wild rocket decreased the relative abundance of Actinobacteria and increased that of Acidobacteria in cucumber rhizosphere (*p <* 0.05).

At the genus level, more than 660 bacterial genera were detected. For dominant classified bacterial genera (average relative abundances >0.50% in at least one treatment), rotations with Indian mustard and wild rocket increased the relative abundances of *Nitrospira*, *Opitutus*, *Pirellula* spp. and decreased these of *Lysobacter*, *Streptomyces*, *Pseudoduganella*, *Nocardioides* and *Agromyces* spp. (*p <* 0.05) (Table 2). Moreover, rotation with Indian mustard decreased the relative abundance of *Rhodanobacter* and *Novosphingobium* spp., and increased these of *RB41* and *Archangium* spp. in cucumber rhizosphere (*p <* 0.05). Rotation with wild rocket decreased the relative abundances of *Bacillus* and *Turicibacter* spp. in cucumber rhizosphere (*p <* 0.05). *Pseudomonas* spp. was detected at relative low abundance (average relative abundance was 0.35% across all samples). The average relative abundances of *Pseudomonas* spp. in treatments of rotations with Indian mustard (0.38 ± 0.06%) and wild rocket (0.45 ± 0.06%) were higher than in the monocropping treatment (0.23 ± 0.03%) (*p* *<* 0.05).

### 3.5. Correlation between Cucumber Seedling Biomass, Fusarium Wilt Disease Severity and Bacterial Taxa Abundance

Cucumber seedling biomass was positively correlated with the relative abundances of *Lysobacter*, *Nocardioides*, *Rhodanobacter*, *Phenylobacterium* and *Agromyces* spp., and negatively correlated with those of *Nitrospira*, *Opitutus*, *Pirellula* and *Archangium* spp. in cucumber seedling rhizosphere (*p <* 0.05) (Table 3).

Cucumber seedling Fusarium wilt disease severity was positively correlated with the relative abundances of *Clostridium sensu stricto 1*, *Streptomyces*, *Pseudoduganella* and *Bacillus* spp., and was negatively correlated with those of *Nitrospira*, *RB41* and *Pirellula* spp. in cucumber seedling rhizosphere (*p <* 0.05).

## 4. Discussion

Cucumber Fusarium wilt disease is one of the most devastating soil-borne fungal diseases in cucumber production [27]. Previous research indicated that Fusarium wilt diseases tend to be less susceptible to the direct effects of biofumigation than most other pathogens, such as *Sclerotium cepivorum* and *Rhizoctonia solani* [17,21]. Here, our results showed that rotations with Indian mustard and wild rocket suppressed cucumber Fusarium wilt disease, which was in line with previous studies showing that rotations with Brassicaceae crops (with no incorporation of aboveground biomass) can suppress plant soil-borne diseases [21,22,23]. Antifungal compounds released by Brassicaceae crops (*e.g.,* isothiocyanates) have been mentioned as a major causal factor to inhibit soil-borne plant pathogens [1]. However, these compounds usually have short half-life time (only a few days) in the soil environment [40,41]. In our experiment, roots of Indian mustard and wild rocket had been decomposed for 45 days in soil when cucumber seedlings were challenged with FOC. Therefore, direct inhibition activity of antifungal compounds from roots of Indian mustard and wild rocket might play a minor role in suppressing cucumber Fusarium wilt disease in this study. However, direct inhibition could play a major role in situations where soil-borne plant pathogens were inoculated before Brassicaceae crops were planted as observed in other studies [1,21,40].

Plant rhizosphere microorganisms play pivotal roles in modulating plant growth and health [8]. In this study, rotations with Indian mustard and wild rocket changed cucumber rhizosphere bacterial community composition, and increased bacterial diversity and abundance, which validated results of previous studies [4,5,6,7,16,22]. Moreover, rotations with Indian mustard and wild rocket increased *Pseudomonas* spp. and DAPG producer abundances. Some species in *Pseudomonas* spp. and their secondary metabolites, including DAPG, can protect plants directly by inhibiting plant pathogens and indirectly by inducing systemic resistance in plants [9,10]. Previous studies also showed that increasing microbial abundance and diversity can inhibit the invasion of pathogens by competing for space and resources [42,43]. Thus, the decreased cucumber Fusarium wilt disease severity and FOC abundance may be linked to the increased bacterial community diversity and abundances of total bacteria and specific microbial taxa with antifungal activities in the rotation treatments.

Rotations with Indian mustard and wild rocket increased the relative abundance of *Opitutus* spp. in cucumber rhizosphere. Moreover, cucumber seedling Fusarium wilt disease severity was negatively correlated with the relative abundance of *Pirellula* spp. in cucumber seedling rhizosphere. These results were in line with previous studies showing that abundance of *Pirellula* spp. was negatively correlated with cucumber and Lanzhou lily Fusarium wilt disease severities [44,45]. Therefore, it is possible that *Opitutus* spp. may contain species with antifungal activities and further studies should be done to isolate strains of *Opitutus* spp. and test their antifungal activities *in vitro*.

The relative abundances of some bacterial genera rich in strains associated with plant-growth-promoting and/or plant pathogen-inhibiting potentials in cucumber rhizosphere were inhibited by rotations with Indian mustard and wild rocket. For example, rotations with Indian mustard and wild rocket decreased the relative abundances of *Lysobacter* and *Streptomyces* spp. [46]. Rotation with wild rocket decreased the relative abundance of *Bacillus* spp. [10]. However, previous studies found that green manures or amendments of seed meals of Brassicaceae crops could promote these bacterial genera [47,48]. One explanation for these inconsistencies is that the soil used in this study contained bacterial taxa that were sensitive to secondary compounds released by Brassicaceae crops. It has been shown that isothiocyanates had inhibitory effects on many bacterial strains, including *Bacillus* spp. [49]. Moreover, it was usually observed that the response of soil microbial communities could differ with types of plant materials used to amend as different types of plant materials had different chemistry compositions (types and concentrations of isothiocyanates) [1,12,50] and different isothiocyanates could exert different influences on soil microbial communities [41]. Another explanation is that different parts of plant materials were decomposed in the soil. For example, plant aboveground and belowground residues had different chemical composition and could exert different effects on soil microbial communities [40,51]. However, data from Illumina Miseq sequencing referred to relative abundances and the rotations increased the richness and diversity of rhizosphere bacteria community (meaning more and a wider variety of bacteria were present), that could mean that the relative abundances of *Bacillus*, *Lysobacter* and *Streptomyces* spp. only decreased in relative abundance due to the increases in many other organisms, and were not necessarily due to an actual decrease in numbers.

Here, we found that rotations with Indian mustard and wild rocket decreased soil phenolic compounds. Autotoxic compounds (such as phenolic compounds) released from crops residues and rhizodeposition can promote the proliferation of soil pathogens and decrease soil bacterial diversity [28,52,53,54]. Therefore, the increased bacterial community diversity and decreased FOC abundance in cucumber rhizosphere might be linked to the decreased soil phenolic compounds in the rotation treatments. It has been demonstrated that increasing plant diversity, including plant litter diversity, can enhance plant litter decomposition [55]. It was possible that rotations with Indian mustard and wild rocket increased cucumber root decomposition and autotoxic compounds releasing rates, which warrants further investigations.

Previously, we observed that green manures of Indian mustard and wild rocket suppressed cucumber Fusarium wilt disease and promoted cucumber growth. Here, our results showed that rotations with Indian mustard and wild rocket without their aboveground materials incorporated into soil suppressed cucumber Fusarium wilt disease but inhibited cucumber seedling growth. Since no fertilizer was added in this experiment and aboveground materials of Indian mustard and wild rocket were removed and not incorporated into the soil, it was not surprising that rotations with Indian mustard and wild rocket decreased soil inorganic N content. It was possible that the depletion of soil nutrients by Indian mustard and wild rocket was responsible for the decreased cucumber seedling growth in treatments of rotations with Indian mustard and wild rocket. Rotations with Indian mustard and wild rocket decreased the relative abundances of *Agromyces* [56], *Lysobacter* [57], *Nocardioides* [58] and *Streptomyces* spp. [59], whose species can have plant-growth-promoting effects. Therefore, another possible explanation for the decreased cucumber seedling biomasses was rotations with Indian mustard and wild rocket reduced some plant-beneficial microorganisms. Other agricultural management practices (such as N fertilization rate) should be optimized in the rotation systems of Indian mustard and wild rocket with cucumber to make these rotation systems promoting cucumber growth and more efficacious against cucumber Fusarium wilt.

## 5. Conclusions

In summary, this study found that rotations with Indian mustard and wild rocket suppressed cucumber Fusarium wilt disease and changed rhizosphere bacterial community composition. Rotations with Indian mustard and wild rocket increased bacteria diversity and abundances of *Pseudomonas* spp. and DAPG producer in cucumber rhizosphere, which may contribute to decreased FOC abundance in cucumber rhizosphere. Our study stressed the view that it is feasible to harnessing crop rhizosphere microbiome through diversified cropping systems to control plant diseases [60]. However, rotations with Indian mustard and wild rocket inhibited cucumber seedling growth and decreased soil inorganic N contents. It should be noted that our results were relatively tentative (based on one experiment only) and further experimental repetitions are needed in order to make substantiated conclusions.

## Figures and Tables

**Table 1:** Effects of crop rotations on cucumber seedling dry biomass, Fusarium wilt disease index, and soil chemical properties.

	Cucumber Dry Biomass (g/plant)	Fusarium Wilt Disease Index (%)	Soil pH	Soil Olsen P (mg/kg)	Soil Inorganic N (mg/kg)	Soil Phenolic Compounds (μg Ferulic Acid/g Soil)
C ^1^	8.06 ± 0.32 a ^2^	66.49 ± 1.41 a	7.64 ± 0.05 a	284.20 ± 7.91 a	146.59 ± 7.10 a	217.01 ± 7.75 a
M	5.06 ± 0.48 b	49.50 ± 1.87 b	7.45 ± 0.08 a	266.91 ± 6.62 a	114.09 ± 3.85 b	169.05 ± 11.31 b
WR	5.46 ± 0.46 b	48.46 ± 4.51 b	7.43 ± 0.07 a	265.03 ± 5.25 a	119.98 ± 4.15 b	172.76 ± 5.34 b

^1^ C, M and WR represent treatments of cucumber monocropping, rotations with Indian mustard and wild rocket, respectively. ^2^ Different letters indicate statistically significant differences among treatments (Tukey’s HSD, *p <* 0.05).

**Table 2:** Relative abundances (%) of main bacterial genera in cucumber rhizosphere soils. Bacterial genera with average relative abundances >0.50% in at least one treatment were shown.

	C ^1^	M	WR		C	M	WR
*Clostridium sensu stricto* ^1^	5.84 ± 0.24 a ^2^	5.68 ± 0.07 a	4.49 ± 1.26 a	*H16*	0.57 ± 0.06 a	0.64 ± 0.03 a	0.83 ± 0.08 a
*Gaiella*	2.21 ± 0.35 a	1.82 ± 0.17 a	1.69 ± 0.16 a	*RB41*	0.42 ± 0.05 b	0.74 ± 0.05 a	0.70 ± 0.10 ab
*Terrisporobacter*	1.65 ± 0.13 a	1.74 ± 0.12 a	1.18 ± 0.29 a	*Microlunatus*	0.63 ± 0.02 a	0.64 ± 0.05 a	0.49 ± 0.09 a
*Steroidobacter*	1.39 ± 0.15 a	1.63 ± 0.02 a	1.45 ± 0.10 a	*Pedomicrobium*	0.53 ± 0.03 a	0.61 ± 0.04 a	0.50 ± 0.01 a
*Acidibacter*	1.49 ± 0.19 a	1.44 ± 0.09 a	1.47 ± 0.11 a	*Rhodanobacter*	0.72 ± 0.02 a	0.30 ± 0.05 b	0.59 ± 0.04 a
*Nitrospira*	0.88 ± 0.03 b	1.19 ± 0.05 a	1.18 ± 0.01 a	*Opitutus*	0.31 ± 0.03 b	0.72 ± 0.07 a	0.57 ± 0.05 a
*Lysobacter*	1.40 ± 0.09 a	0.74 ± 0.06 b	1.06 ± 0.07 b	*Pir4 lineage*	0.47 ± 0.03 a	0.59 ± 0.08 a	0.52 ± 0.05 a
*Streptomyces*	1.58 ± 0.27 a	0.64 ± 0.07 b	0.66 ± 0.13 b	*Pirellula*	0.39 ± 0.01 b	0.57 ± 0.05 a	0.54 ± 0.03 a
*Actinoplanes*	0.98 ± 0.08 a	1.02 ± 0.22 a	0.78 ± 0.09 a	*Sporosarcina*	0.55 ± 0.02 a	0.54 ± 0.03 a	0.38 ± 0.09 a
*Gemmatimonas*	0.98 ± 0.08 a	0.91 ± 0.07 a	0.89 ± 0.03 a	*Phenylobacterium*	0.54 ± 0.02 a	0.38 ± 0.05 a	0.47 ± 0.06 a
*Haliangium*	0.69 ± 0.03 a	0.91 ± 0.11 a	0.85 ± 0.03 a	*Roseiflexus*	0.41 ± 0.04 a	0.54 ± 0.07 a	0.42 ± 0.03 a
*Pseudoduganella*	1.67 ± 0.15 a	0.37 ± 0.02 b	0.32 ± 0.02 b	*Novosphingobium*	0.59 ± 0.08 a	0.23 ± 0.04 b	0.46 ± 0.04 ab
*Nocardioides*	1.02 ± 0.10 a	0.57 ± 0.02 b	0.68 ± 0.03 b	*Agromyces*	0.58 ± 0.05 a	0.34 ± 0.03 b	0.36 ± 0.03 b
*Bacillus*	0.91 ± 0.04 a	0.79 ± 0.04 ab	0.49 ± 0.11 b	*Chryseolinea*	0.42 ± 0.02 a	0.30 ± 0.07 a	0.53 ± 0.06 a
*Turicibacter*	0.86 ± 0.13 a	0.84 ± 0.02 ab	0.48 ± 0.06 b	*Solirubrobacter*	0.51 ± 0.11 a	0.36 ± 0.05 a	0.36 ± 0.02 a
*Bryobacter*	0.65 ± 0.04 a	0.79 ± 0.05 a	0.67 ± 0.01 a	*Archangium*	0.28 ± 0.05 b	0.62 ± 0.12 a	0.32 ± 0.04 ab

^1^ C, M and WR represent treatments of cucumber monocropping, rotations with Indian mustard and wild rocket, respectively. ^2^ Values (mean ± SE) for each genus within rows with different letters are significantly different (Tukey’s HSD, *p <* 0.05).

**Table 3:** Spearman correlations between cucumber seedling biomass (CSB), Fusarium wilt disease severity (FWDS) and main classified bacterial taxa (average relative abundances >0.50% in at least one treatment).

	CSB	FWDS		CSB	FWDS
*Clostridium sensu stricto 1*	0.17	0.68 ^1^	*H16*	−0.61	−0.28
*Gaiella*	0.58	0.22	*RB41*	−0.58	**−0.83**
*Terrisporobacter*	−0.08	0.36	*Microlunatus*	−0.10	0.43
*Steroidobacter*	−0.52	−0.55	*Pedomicrobium*	−0.32	−0.05
*Acidibacter*	−0.08	−0.13	*Rhodanobacter*	**0.68**	0.66
*Nitrospira*	**−0.67**	**−0.69**	*Opitutus*	**−0.78**	−0.56
*Lysobacter*	**0.78**	0.54	*Pir4 lineage*	−0.60	0.13
*Streptomyces*	0.65	**0.82**	*Pirellula*	**−0.78**	**−0.76**
*Actinoplanes*	0.28	0.27	*Sporosarcina*	0.35	0.43
*Gemmatimonas*	0.43	−0.15	*Phenylobacterium*	**0.75**	0.13
*Haliangium*	−0.47	−0.63	*Roseiflexus*	−0.40	−0.30
*Pseudoduganella*	0.57	**0.71**	*Novosphingobium*	0.60	0.41
*Nocardioides*	**0.83**	0.59	*Agromyces*	**0.88**	0.53
*Bacillus*	0.46	**0.75**	*Chryseolinea*	0.21	0.02
*Turicibacter*	0.08	0.27	*Solirubrobacter*	0.54	0.10
*Bryobacter*	−0.43	−0.17	*Archangium*	**−0.69**	−0.37

^1^ Values in bold are significant (*p <* 0.05).
